# Prevalence of Alcohol Consumption and Hazardous Drinking, Tobacco and Drug Use in Urban Tanzania, and Their Associated Risk Factors

**DOI:** 10.3390/ijerph6071991

**Published:** 2009-07-16

**Authors:** Joseph Mbatia¹, Rachel Jenkins, Nicola Singleton, Bethany White

**Affiliations:** 1 Director for Mental Health, Ministry of Health, Tanzania; E-Mail: jmbatia@muchs.ac.tz; 2 Director, WHO Collaborating Centre (Mental Health), Institute of Psychiatry, Kings College London, UK; 3 Director of Policy & Research, UK Drug Policy Commission; UK; E-Mail: nicola.singleton@homeoffice.gsi.gov.uk; 4 Research worker, WHO Collaborating Centre (Mental Health), Institute of Psychiatry, Kings College London, UK; E-Mail: bwhite@nchecr.unsw.edu.au

**Keywords:** alcohol, substance use, Tanzania

## Abstract

Evidence suggests substance abuse in Tanzania is a growing public health problem. A random sample of 899 adults aged 15–59 in two urban sites of differing levels of poverty surveyed alcohol, tobacco and illicit substance use. Rates of substance use were 17.2%. 8.7% and 0.8% for alcohol, tobacco and cannabis, respectively. Living in the less affluent area was associated with higher lifetime rates of tobacco and alcohol use. Substance use is less prevalent in Tanzania than in richer countries, but lifetime consumption is higher in poorer areas. The association of substance use with a range of socio-economic factors warrants further research.

## 1. Introduction

Alcohol consumption is an important contributor to the global burden of disease, responsible for 4% of disability-adjusted life years [1,2]. While disease burden is somewhat lower in low income geographic regions where abstinence is common, there is concern that urbanisation is associated with increased alcohol use [3–5].

In Tanzania studies of traditionally brewed beverages [5–7] and of overall alcohol consumption of young people [8,9] use of other drugs [10,11] and heroin users [12,13], have been undertaken, but there have been few community-based studies on alcohol consumption among the general adult population [14–16], while small sample sizes [14,16] and lack of methodological detail limit the generalisability of findings from the studies that have been undertaken..

Problematic alcohol use is associated with economic disadvantage in both resource-rich [17,18] and resource-poor [3,4] countries. The Tanzanian Participatory Poverty Assessment [19] recognised the linkages between poverty and overall ill health, but did not explicitly consider the role of alcohol, tobacco and illicit substance use, which may be crucial facts in linking economic and social development and research on these linkages may assist effective interventions.

This paper therefore used the opportunity of a wider epidemiological survey of psychiatric morbidity in an urban surveillance site in Tanzania to examine: (i) the extent of alcohol use and hazardous drinking, tobacco and (ii) the relationships between hazardous alcohol use, use of other substances, socio-demographic factors, (including poverty) and economic and social functioning.

## 2. Methods

### 2.1. Sites

This study is a cross-sectoral study conducted across two sites. In September and October 2003 a population-based survey was conducted in two urban areas of Dar es Salaam, Tanzania’s largest city, with a population of 2.5 million. The areas were sites of the Adult Morbidity and Mortality Project (AMMP) [20,21], selected to ensure subpopulations of differing socio-economic circumstances. Ilala-Ilala (Ilala municipality) was a middle-income area of government-built housing where small business owners and office workers from diverse ethnic backgrounds resided, while Mtoni-Saba Saba (Temeke municipality) was a low-income area where traders and farmers lived in high-density housing [22]. In 2002, the populations of the Ilala and municipalities were 634,924 and 768,451 respectively [23].

### 2.2. Sample

As the population in the two geographically defined areas was enumerated regularly as part of the AMMP, it was possible to draw a sample of individuals thus avoiding the usual issue of clustered sample designs. A systematic sample of 1,100 adults aged 15–59 was drawn from a random starting point from the AMMP lists (ordered by age within household); 550 from the eligible population of 4,690 in Ilala-Ilala, and 550 from an eligible population of 11,620 in Mtoni-Saba Saba [24]. If the person randomly selected for interview had moved away, one of the eligible individual who had moved into the house was interviewed instead (with selection within the household undertaken using the Kish grid method.

### 2.3. Procedures

The Mental Health Section of the Ministry of Health, the Health Research Systems Section of the Directorate of Planning, and Dar es Salaam City Health Services coordinated the survey. Interviews were conducted by volunteer community health workers based in primary health care centres, trained in administration of the pencil and paper interview. Written, informed consent was obtained.

### 2.4. Instruments

Demographic characteristics, socio-economic factors, recent life events and social supports were documented. The Alcohol Use Disorders Identification Test (AUDIT) measured hazardous alcohol use [25] and the Clinical Interview Schedule Revised (CIS-R) [26] assessed common mental disorder (CMD).

Demographic information collected included sex, age, marital status, ethnicity, and household status (head, spouse or other). Socio-economic factors included employment status, income, education attainment, housing tenure (owned or rented) and accommodation type (whole house or room only). We did not collect data on religious affiliation.

Current alcohol use was determined by a positive response to the question ‘*Do you ever drink alcohol nowadays, including drinks you brew or make at home?’* Lifetime non-drinkers (abstainers) were those who answered yes to *‘Have you always been a non-drinker’.* The category of lifetime drinkers included were those who had not always been a non-drinker and current drinkers. Current smokers were those who answered yes to ‘*Do you smoke cigarettes at all nowadays?*’ Participants were considered lifetime smokers if they responded yes to ‘*Have you ever smoked a cigarette?*’ or reported smoking “nowadays”. Participants were then asked whether they had ever used a range of illicit substances including cannabis, amphetamine, cocaine, heroin, ecstasy, LSD, magic mushrooms, and methadone Those who reported lifetime use were asked about use in the previous 12 months.

The AUDIT is a cross-culturally validated instrument for assessment of alcohol misuse in the general population [25]. The ten- item instrument includes questions to determine patterns of drinking considered harmful, hazardous and symptomatic of dependence in the preceding 12 months. Each question is scored between zero and four with a score of eight and over considered indicative of hazardous use.

The CIS-R [26] is a gold standard instrument for use by lay interviewers in assessing common mental disorders in community settings, which has been widely used in low-income countries [27–29], including Tanzania [30]. Scores are calculated from an average of four questions across 14 symptom types and taken together with algorithms based on the ICD-10 [31] provide six possible neurotic diagnoses including depressive episode (mild, moderate or severe), obsessive compulsive disorder, panic disorder, phobic disorder, generalised anxiety disorder and mixed anxiety/depressive disorder. However, for the purpose of the current paper, a score of 12 or more across the 14 sections of the survey was considered an indication of any CMD. See Singleton *et al*. [32] for detailed information on scoring.

All respondents were given a list of 18 different stressful life events, and asked to say which, if any, they had experienced in the past six months. The list included relationship problems, employment, financial crises and victimisation experiences. The list was developed for the 1993 British Psychiatric Morbidity Survey [33,34], and tailored for the Tanzania context. For the purposes of analysis, life event scores were grouped into no life events, one, two and three or more life events.

Perceived social support was assessed from respondents’ answers to seven questions previously used for the 1992 Health Survey for England [35] and the ONS surveys of psychiatric morbidity [32,36]. The seven questions take the form of statements that individuals could say were not true, partly true or certainly true for them in response to the question ‘There are people I know who’: (i) Do things to make me happy; (ii) Who make me feel loved; (iii) Who can be relied on no matter what happens; (iv) Who would see that I am taken care of if I needed to be; (v) Who accept me just as I am; (vi) Who make me feel an important part of their lives; and (vii) Who give me support and encouragement. Results were categorised into no, moderate or severe lack of perceived social support.

Information on social networks was obtained through questions about the number of friends or relatives who informants felt close to including: (i) Adults who lived with the respondent and to whom they felt close; (ii) Relatives living elsewhere to whom they felt close; and (iii) Friends or acquaintances living elsewhere who informants would describe as close or good friends. These questions were taken from psychiatric morbidity surveys conducted in Britain [37,38] and results grouped none to three, four to eight and nine or more.

### 2.5. Data Analysis

Data were analysed using SPSS software for Windows Version 15 [39]. Chi squared (χ^2^) tests examined demographic and socio-economic differences between the two areas as well as differences in perceived social support and recent life events. Frequencies and cross-tabulations for lifetime, current and recent drug use were computed. Data from the two areas was combined and odd ratios (OR) with 95% confidence intervals (CI) were calculated to determine significant associations with hazardous alcohol use. Univariately significant factors as well as factors significantly different between areas were included in forward step-wise logistic regression and adjusted ORs produced.

### 2.6. Ethics Approval

Approval was granted by the National Institute for Medical Research, Ministry of Health, United Republic of Tanzania and the Ethics Committees of the South London and Maudsley (SLaM), National Health Service (NHS) Foundation Trust.

## 3. Results

### 3.1. Response Rates

Of the 1,100 households approached, 899 residents agreed to participate, giving an overall response rate of 82%. The response rate was slightly higher in Ilala (87%) compared to Saba Saba (76%). The proportion of replacements by new residents when the original person selected for interview no longer resided at the household was not recorded.

### 3.2. Demographic, Socio-Economic and Social Differences between Areas

Respondents from Saba Saba and Ilala were of comparable age (p = 0.51), gender (p = 0.76) and marital status (p = 0.69) but respondents from Ilala were significantly more likely to be household head (p < 0.001), be of non-African ethnicity (p < 0.001) and to report renting their home (p = 0.04), the latter two factors being consistent with census data [24, 40]. Living in poorer Saba Saba was associated with unemployment (p = 0.01) and younger school leaving age (p = 0.06), the direction of this association was again consistent with census data, and participants from Saba Saba reported a significantly higher number of life events in the six months preceding interview (p < 0.001) (see Table 1).

### 3.3. Abstinence

Two thirds (66.9%) of the sample reported lifetime abstinence from alcohol. The rate was significantly higher among women (72.7%), compared to men (59.3%, OR = 1.83 95% CI 1.38–2.43; p < 0.001) and marginally higher in Ilala (69.6%) than Saba Saba (63.6%, OR = 1.31, 95% CI 0.99–1.75; p < 0.06).

### 3.4. Alcohol, Tobacco and Cannabis Use

Table 2 presents prevalence rates for alcohol, tobacco and cannabis use. Rates of current alcohol and tobacco use were 17.2% and 8.7%, respectively, with past-year cannabis use being less common (0.8%). Women were significantly less like than men to report the current use of alcohol (OR = 0.53 95% CI 0.37–0.75, p < 0.001) and tobacco (OR = 0.54 95% CI 0.02–0.12, p < 0.001) and no females reported the use of cannabis in the preceding year (statistical results not shown in Table 1). Sex differences in patterns of lifetime use of these drugs were similar.

The prevalence of both lifetime and recent alcohol and tobacco use was highest among older aged groups while cannabis use was more common among younger people. Those widowed or no longer married had the highest rates of lifetime alcohol use, but married people were more likely to report current use. Single people had higher rates of both lifetime and recent tobacco and cannabis use. The use of all drug types was most common among the employed and older school leavers (Table 2).

Current use of alcohol and tobacco was higher in the poorer area of Saba Saba compared to Ilala although only lifetime rates were significantly different (OR = 0.72; 95% CI 0.54–0.97, p = 0.03; OR = 0.64; 95% CI 0.42–0.97, p = 0.03 respectively). Lifetime and past year cannabis use did not differ by area (statistical results not shown in Table 1). The use of other illicit drugs was rare, with one respondent reporting having ever used cocaine and LSD.

### 3.5. Hazardous Alcohol Use

The prevalence of hazardous alcohol use (AUDIT score greater than 8) was 5.7%. Forward stepwise logistic regression modelling allowed for adjustment of variables significant at the bivariate level including gender, age, household status, employment status, number of recent life events and CIS-R score (CMD), and for differences between areas (ethnicity, housing tenure and education). Hazardous alcohol use remained positively associated with male gender and any CMD and negatively associated with being economically inactive.

## 4. Discussion

This study provides epidemiological information about alcohol use in urban Dar es Salaam, Tanzania, and found that current alcohol and tobacco use was 17.2% and 8.7% respectively and past year cannabis use was 0.8%. The prevalence of hazardous alcohol use was 5.7%. Elevated rates of hazardous drinking were found in males, people aged 25–34, household heads, employed people, those who had experienced more than three life events and those with CMD. Only gender, employment status and CMD remained independently associated with hazardous alcohol use after adjustment for other factors.

To our knowledge this is the first study comparing substance use across two urban areas of differing levels of poverty and housing density in a low income country. Compared to living in Ilala, living in poorer Saba Saba was associated with unemployment, younger school leaving age and reporting more recent life events. While the areas were similar in terms of age, sex and marital status, living in the poorer area was associated with significantly higher lifetime rates of tobacco and alcohol use. Cannabis use and hazardous alcohol use were also higher in Saba Saba although not significantly.

This is also the first study to our knowledge in Sub-Saharan African to report on the relationship between hazardous alcohol use and life events, perceived social support and size of primary support group, and is one of the few studies in Sub-Saharan Africa to report on the relationship between hazardous alcohol use and employment status, income and education, and none have previously reported on the relationship with housing tenure or accommodation type. The sampling frame was well defined and based on biannual demographic surveillance for the AMMP, however, adequate supervision of the implementation of the survey was difficult for logistical reasons and due to budget constraints. This resulted in missing data and a failure to record how often the person randomly selected for interview had moved since the last AMMP census and was therefore replaced by a new resident. Further, the use of measures originally designed for other contexts may have affected the sensitivity of the instruments [41], although instruments previously used in this context were utilised where possible. It should also be noted that self-reported sensitive information such as drug use is prone to social desirability bias and underreporting that may have resulted in systematic measurement error and lowered prevalence estimates. In the regression analysis, data from the two areas were combined without weighting for the different probabilities of selection although variables that differed significantly between areas were included in the modelling of hazardous alcohol use. Also, the same variables were independently associated with hazardous alcohol use in regression models for each area individually (data not shown). Finally, the current findings are specific to the two wards in urban Dar es Salaam and are not necessarily applicable to other parts of Tanzania, particularly rural areas.

### 4.1. Alcohol Abstinence and Use

The prevalence of lifetime abstinence was 66.9%, which was lower than the reported 77.5% of respondents classified as non-drinkers (no alcohol past six months) in all eight of the AMMP census rounds of Dar es Salaam surveillance [22]. When “previous drinkers” (i.e., current non-drinkers) are added to the rate obtained in the current study however, the resulting 72.4% is not dissimilar to the AMMP rate.

The overall prevalence of lifetime alcohol use across two urban areas of Dar es Salaam was 28.9%, with the rate significantly higher among males (38.5%) compared to females (23.7%). The estimates from this study are somewhat lower than results from a community survey of women with partners (n = 1200), women without partners (n = 614) and men (n = 788) in the urban district Moshi in the Kilimanjaro region, where 68.3% males and 50.8 and 57.5% of females with and without partners respectively, had ever used alcohol [15].

Almost one-fifth (17.2%) of respondents reported current drinking. This rate is lower than the respective prevalences of 33.7% and 19.4% of past month drinking in the other urban municipality of Dar es Salaam (Kinondoni) and Old Stone Town (Zanzibar) [11], but consistent with the AMMP census (22.5%) of all surveillance sites in Dar es Salaam [22]. In this study, significantly more males (22.6%) than females (13.3%) were also current drinkers. These rates compare to the results of a survey of Keko ward (n = 246) also in Temeke, where 27.5% of males and 15.7% of females were ‘drinkers’ [13] although again lower than those reported from a rural subdistrict in the Mwanza region where 55% of males (n = 148) and 33% of females (n = 162) had used alcohol at least once in past year [16]. The AMMP also detected higher rates of drinking in rural compared to urban areas and the authors proposed several explanations, including reporting biases related to differences in perception of whether locally brewed beverages constitute alcohol consumption and/or differing economic circumstances between areas [22]. It is likely that there is increased availability of traditional brews in rural areas which are cheaper than proprietary brands, while the necessity to purchase alcohol may be a constraint on use in urban areas.

Our figures are comparable to reports from large community-based surveys in other parts of Sub-Saharan Africa. In Nigeria, 32.7% of males and 5.3% of females reported past year alcohol use [42] while in South Africa, the rate of current alcohol use was 45% among men and 17% of women [43]. In contrast, on older study by Rahim and Cederblad [44] in the Sudan found that alcohol abuse was very rare (0.4%) with no sex differences in the prevalence.

### 4.2. Hazardous Alcohol Use

In this study, males (8.7%) were significantly more likely than females (3.4%) to report hazardous alcohol use. In urban Moshi (Kilimanjaro Region), Mitsunga and Larsen [15] found significantly higher rates of alcohol abuse among males (22.8%) than females with (7.0%) and without (9.3%) partners according to the CAGE questionnaire while males in the rural subdistrict of the Mwanza Region drank higher quantities more frequently in the preceding six months compared to females [16]. Higher rates of problematic alcohol use among males have been reported both in other regions of sub-Saharan Africa [42,43] and other regions of the world [17,18]. Despite men being at greater risk, women also face negative alcohol-related consequences in Tanzania [45], Africa [46] and elsewhere [47]. As in South Africa, (e.g., 43), women in Tanzania may under report alcohol and substance abuse.

Similar to results from the UK [32], where those with no hazardous pattern of alcohol use were most likely to be economically inactive (unemployed but not seeking work), the economically inactive in Tanzania had significantly lower rates of hazardous use, possibly as a result of a reduced capacity to purchase alcohol. Consistent with this, those with an income had slightly higher rates of alcohol, tobacco and cannabis use. Conversely, the unemployed group had the highest prevalence for hazardous drinking, again consistent with evidence from the UK [32]. Finally, the association with common mental disorder and hazardous alcohol use is well established in developed countries [48–50] and other low income geographic regions [51,52] but whether CMD causes hazardous alcohol consumption or whether drinking at harmful levels increases the risk of CMD cannot be determined from the current data.

### 4.3. Associations with Other Socio-Demographic Variables

In the current study, the peak age for hazardous alcohol consumption was 25–34, similar to the Sub-Sahara African studies [42,43] and in contrast to a much wealthier country such as Britain, where the peak age is 16–19 in women and 20–24 in men [32]. These peak age differences may reflect access to money and the ability to buy alcohol, as well as social norms and parental restrictions on the acceptability of hazardous alcohol consumption in young people. People aged 25–34 were also most likely to report current tobacco use whereas the peak age for cannabis use was 16–24.

In Dar es Salaam, single people had higher rates of hazardous alcohol consumption, whereas in Britain, rates were substantially higher in separated, widowed and divorced individuals of both sexes [53]. It may be that in Tanzania, there is a greater capacity to pay for alcohol in single people than in those who are separated, divorced or widowed, an economic factor which may override the usual increased vulnerability of the separated, divorced or widowed. At the same time, single people in Tanzania overall had the lowest rates of lifetime and current alcohol use and highest rates of tobacco and cannabis use, perhaps an indication of age, but suggesting that patterns of substance use among this group require further investigation.

There was no clear relationship between education and hazardous alcohol use although those leaving school at 19 or over had slightly higher rates that may reflect purchasing capacity and opportunity. Older school leaving age groups also had the highest rates of alcohol, tobacco and cannabis use.

Indigenous Africans had the highest prevalence rates; other ethnic groups were not drinking. This could perhaps be a reflection of differing religious beliefs, which were not documented in the current study.

In Tanzania household heads were significantly more likely to report hazardous alcohol use although the association did not remain significant after adjustment for other factors. It may also reflect greater access to money. Those living rent free had highest rates of hazardous drinking across which may be a reflection of a higher disposable income, whereas in the British survey, hazardous drinking was highest among those with a mortgage [32].

We were unable to identify a previous Sub-Saharan Africa study of the relationship of hazardous alcohol use with life events, perceived social support and size of primary support group. Higher rates of hazardous drinking were observed among those who had experienced more than three life events in the six months preceding interview and those with a severe lack of social support compared to those with no lack. Interestingly however, those with a primary support group of more than four people had the higher rates of hazardous drinking compared to those with a primary support group of three or less. It seems that in Tanzania, size of social network has a different effect than strength of perceived social support.

## 5. Conclusions

Substance use is less prevalent in Tanzania than in richer countries, but lifetime consumption is significantly higher in poorer areas. Hazardous alcohol consumption was unaffected by the poverty disparities of the two geographic areas, but was independently associated with male gender, employment status and CMD. Further longitudinal research is needed to tease out the direction of causal linkages.

## Figures and Tables

**Table 1.** Demographic & socio-economic factors, social support and life events by area.

	**Saba Saba n = 418 (%)**	**Ilala n = 481 (%)**	**P value (χ^2^)**
**Gender**			
Male Female	44 56	43 57	0.760
**Age**			
16–24 25–34 35+	32 34 34	29 34 37	0.508
**Marital status**			
Married/cohabitating Single Widowed/divorced/separated	55 38 8	56 36 9	0.687
**Relationship to household head**			
Head Spouse/partner Other	35 32 34	45 33 23	0.000
**Ethnic group**			
Black African Other	98 2	88 12	0.000
**Employment status**			
Working Unemployed Economically inactive	32 9 59	39 3 58	0.000
**Own/rent accommodation**			
Owns Rents Rent free	49 48 2	41 55 4	0.036
**Type of accommodation**			
Whole house Rooms/flat/other	46 54	41 59	0.107
**Age left full time education**			
13 or under/Never went 14–16 17 or 18 19+yrs Still at school	8 38 24 20 10	5 38 24 27 6	0.012
**Income**			
Yes No	44 55	41 59	0.349
**Perceived social support**			
Severe lack Moderate lack No lack	21 37 41	21 34 44	0.708
**Size of Primary Social support group**			
0–3 4 to 8 9 or more	14 42 44	15 49 36	0.408
**Number of life events**			
None 1 2 3+	56 26 12 7	72 21 6 3	0.000

**Table 2.** Prevalence of alcohol, tobacco and cannabis use by demographic and socioeconomic characteristics.

		**Alcohol**		**Tobacco**		**Cannabis**	
**Characteristic**	**Sample size**	**Lifetime**	Current*	**Lifetime**	Current*	**Lifetime**	**Past year**
		n (%)	n (%)	n (%)	n (%)	n (%)	n (%)
**Gender**							
Male Female	393 506	146 (38.5) 114 (23.7)	88 (22.6) 67 (13.3)	95 (24.3) 8 (1.6)	72 (18.3) 6 (1.2)	11 (2.8) 1 (0.2)	7 (1.8) –
**Age**							
16–24 25–34 35+	275 308 316	44 (16.7) 160 (35.1) 110 (37.3)	27 (9.9) 67 (21.8) 61 (19.4)	19 (6.9) 41 (13.3) 43 (13.6)	14 (5.1) 33 (10.7) 31 (9.8)	6 (2.2) 3 (1.0) 3 (0.9)	4 (1.5) 1 (0.3) 2 (0.6)
**Marital Status**							
Married/cohabitating Single Widowed/divorced/separated	495 327 75	156 (31.5) 79 (24.2) 25 (33.3)	93 (18.8) 50 (5.3) 12(16.0)	52 (10.5) 41 (12.5) 9 (12.0)	38 (7.7) 32 (9.8) 7 (9.3)	4 (0.8) 8 (2.4) –	1 (0.2) 6 (1.8) –
**Employment status**							
Working Unemployed Economically inactive	300 49 496	128 (42.7) 21 (42.9) 95 (19.2)	77 (25.7) 12 (24.5) 57 (11.5)	63 (21.0) 8 (16.3) 26 (5.2)	50 (16.7) 5 (10.2) 17 (3.4)	9 (3.0) – 3 (0.6)	5 (1.7) – 2 (0.4)
**Age left full time education**							
13 or under/Neverwent 14–16 17 or 18 19+ yrs Still at school	52 337 212 208 71	10 (19.2) 92 (27.3) 61 (28.8) 89 (42.8) 6 (8.5)	7 (13.5) 54 (16.0) 36 (17.0) 53 (25.5) 4 (5.6)	5 (9.6) 30 (8.9) 29 (13.7) 34 (16.3) 4 (5.6)	1 (1.9) 22 (6.5) 26 (12.3) 25 (12.0) 3 (4.2)	– 4 (1.2) 5 (2.4) 2 (1.0) 1 (1.4)	– 2 (0.6) 4 (1.9) – 1 (1.4)
**Area**							
Saba Saba Ilala	418 481	136 (32.5) 124 (28.5)	81 (19.4) 74 (15.4)	58 (13.9) 45 (9.4)	42 (10.0) 36 (7.5)	7 (1.7) 5 (1.0)	6 (1.4) 1 (0.2)
**TOTAL**	**899**	260 (28.9)	155 (17.2)	103 (11.5)	78 (8.7)	12 (1.3)	7 (0.8)

* Current use – participants who responded yes to “do you ever drink or smoke now days”.

**Table 3.** Prevalence and odds ratios for hazardous alcohol use by area.

	**Sample size**	**Number of cases**	**Prevalence (%)**	**Unadjusted odds ratio**	**Adjusted odds ratio**
**Gender**					
Male Female	393 506	34 17	8.7 3.4	1.00 0.37 (0.20–0.67)	1.00 0.51 (0.27–0.98)
**Age**					
16–24 25–34 35+	275 308 316	10 26 15	3.6 8.4 4.7	1.00 2.44 (1.16–5.16) 1.32 (0.58–2.99)	
**Marital Status**					
Married/cohabitating Single Widowed/divorces/separated	495 327 75	27 22 2	5.5 6.7 2.7	1.00 1.25 (0.70–2.24) 0.47 (0.11–2.04)	
**Relationship to household head**					
Head Spouse/cohabitating Other	359 290 250	29 10 12	8.1 3.4 4.8	1.00 0.41 (0.19–0.85) 0.57 (0.29–1.15)	
**Ethnic group**					
Black African Other	834 63	51 0	6.1 0.0	–	
**Employment status**					
Working Unemployed Economically inactive	300 49 496	30 8 13	10.0 16.3 2.6	1.00 1.76 (0.75–4.09) 0.24 (0.12–0.47)	1.00 1.67 (0.69–4.11) 0.30 (0.15–0.61)
**Own/rent accommodation**					
Owns Rents Rent free	403 463 29	22 25 4	5.5 5.4 13.8	1.00 0.99 (0.55–1.78 2.77 (0.89–8.66)	
**Type of accommodation**					
Whole house Rooms/flat/other	386 510	24 27	6.2 5.3	1.00 0.84 (0.48–1.49)	
**Age left full time education**					
13 or under/Never went 14–16 17 or 18 19+ yrs Still at school	52 337 212 208 71	2 17 12 18 2	3.8 5.0 5.7 8.7 2.8	1.00 1.33 (0.30–5.92) 1.50 (0.33–6.92) 2.37 (0.53–10.55) 0.72 (0.10–5.32)	
**Income**					
Yes No	354 476	24 24	6.8 5.0	1.00 0.73 (0.41–1.31)	
**Perceived social support**					
Severe lack Moderate lack No lack	173 288 341	13 16 18	7.5 5.6 5.3	1.00 0.72 (0.34–1.54) 0.69 (0.33–1.43)	
**Size of Primary Social support group**					
0–3 4 to 8 9 or more	130 411 358	4 30 17	3.1 7.3 4.7	1.00 2.48 (0.86–7.18) 1.57 (0.52–4.76)	
**Number of life events**					
None 1 2 3+	576 206 76 41	27 13 5 6	4.7 6.3 6.6 14.6	1.00 1.37 (0.69–2.71) 1.43 (0.53–3.84) 3.49 (1.35–9.00)	
**CIS- R (CMD)**					
<12 12 +	872 27	46 5	5.3 18.5	1.00 4.08 (1.48–11.27)	1.00 4.34 (1.44–13.10)
**Area**					
Saba Saba Ilala	418 481	26 25	6.2 5.2	1.00 0.83 (0.47–1.45)	
